# Measuring Liquid Drop Properties on Nanoscale 1D Patterned Photoresist Structures

**DOI:** 10.1038/s41598-019-42106-z

**Published:** 2019-04-05

**Authors:** Juan J. Faria-Briceno, Alexander Neumann, P. Randall Schunk, S. R. J. Brueck

**Affiliations:** 1 Center for high Technology and Materials and Department of Electrical and Computer Engineering, University of New Mexico, Albuquerque, 1313 Goddard St. SE, Albuquerque, New Mexico 87106 USA; 2 Department of Chemical and Biochemical Engineering, University of New Mexico, Albuquerque, NM 87131 USA; 3 Advanced Materials Laboratory, Sandia National Laboratories, Albuquerque, NM 87106 USA

## Abstract

This communication reports liquid wetting properties of DI-water on one-dimensional nano-patterned photoresist lines atop a silicon substrate as the pattern period is varied from 0.3- to 1.0-µm. Both constant photoresist height and constant width/height ratios are investigated. The line/period ratio was fixed at 0.3 (0.4) for different measurement sequences. The surface of the photoresist was treated with a short CHF_3_ reactive ion etch to ensure consistent hydrophobic photoresist: water surface energies. Average parallel contact angle (*θ*_||_), average perpendicular contact angle (*θ*_⊥_), drop width (*W*), and drop length (*L*) at constant volume were measured on nano-patterned surfaces fabricated with interferometric lithography. Both *θ*_||_ and *θ*_⊥_ contact angles increase as the period (0.3- to 1-μm) increases; the *θ*_||_ spreading rate is faster than *θ*_⊥_ due to pinning on the grooves resulting in an elongated drop shape. The traditional Wenzel and Cassie-Baxter models of drop contact angles were developed for isotropic random 2D roughness and do not account for the anisotropy induced by the 1D line patterns. The observed angular variations with period are not consistent with either model. Understanding liquid wetting properties and hydrophobicity on 1D silicon surfaces has many applications in lab-on-a-chip, micro/nano-fluidic devices, roll-to-roll nano-imprint fabrication, self-cleaning surfaces, and micro-reactors.

## Introduction

The interaction of liquid drops with patterned surfaces has both intrinsic scientific interest as a result of the complex three-phase interface, which is impacted by both chemical and structural variations of the surface, and technological interest as it impacts diverse contemporary topics such as lab-on-a-chip biosensors, nanoimprint lithography, self-cleaning surfaces and water shedding. The ability and extent to which a liquid wets a surface has always been of paramount importance in manufacturing processes such as solution/spin-on thin-film coating and related processing flows, and the rapid growth in importance of nano-manufacturing processes such as micro-gravure printing^1^ and imprint lithography^2^ are leading to an increasing need to manipulate and position liquid droplets on nano-structured surfaces Microfluidic, lab-on-a-chip devices are of increasing technological interest and require control of the wetting properties on nano-patterned surfaces. Moreover, opportunities exist to exploit macroscopic drop attributes (dimensions/contact angles/rolling resistance) to probe the nanoscale details of the surface, providing a powerful nano-metrology capability that will find many uses in nano-manufacturing.

**Figure 1:**
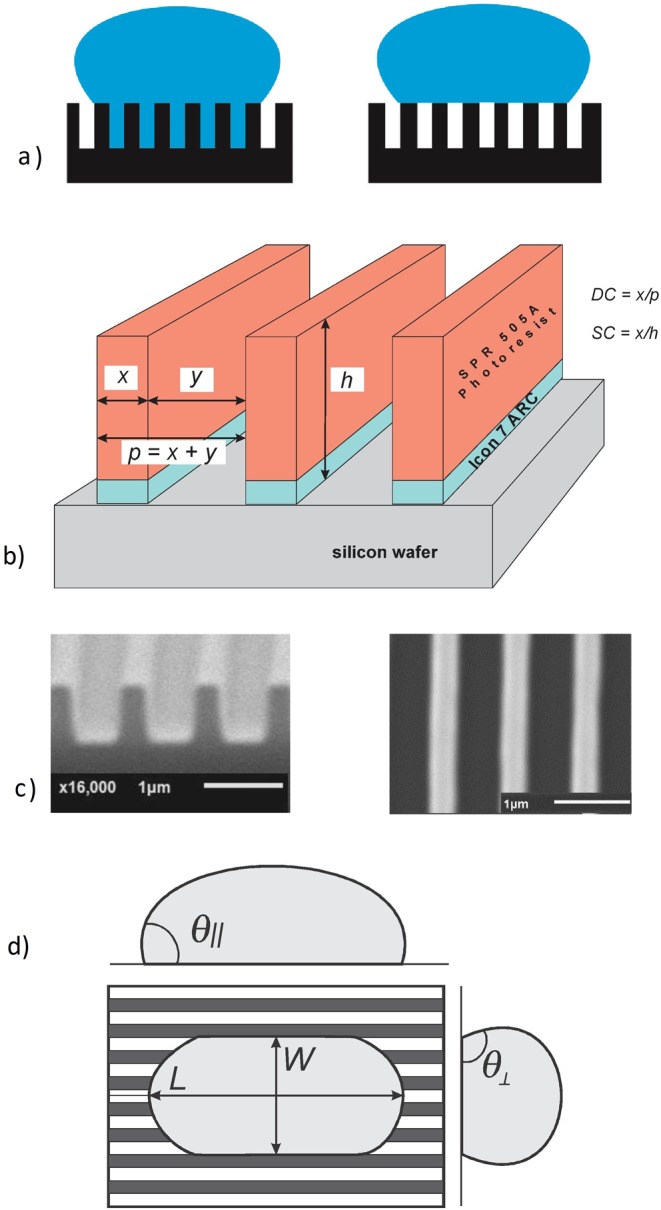
(**a**) In a Wenzel model the liquid conforms to and wets the convoluted surface. For a Cassie-Baxter model, the liquid only contacts the top of the photoresist lines with vapor filling the inside of the troughs. (**b**) Schematic of the 1D photoresist on silicon structure. (**c**) Cross SEM image of 1D positive photoresist (SPR-505A) - 1000-nm period with ~31% *DC*, ~27% *SC*; Top-down SEM image of 1D positive photoresist (SPR-505A) 1000-nm period with ~30% *DC*, ~27% *SC*. (**d**) Definitions of the experimentally reported drop parameters.

The measurement and analysis of contact angles of water on randomly rough 2D surfaces has a long history. Both chemically^3,4^ and structurally^5–9^ inhomogeneous surfaces have been investigated. The contact angle models of Cassie-Baxter (gas trapped under the liquid) and Wenzel (liquid filling the rough surface contour) as a function of the surface roughness have been discussed extensively and are shown schematically in Fig. 1a^10–13^.

With advances in nano-patterning^14^, increasing attention is being given to well characterized surface structures of both 1D (lines)^15–19^, and 2D (arrays of holes/posts) geometries at nanoscale dimensions^20,21^. The formation and dynamics of faceted droplets on larger-scale (>100 μm linear dimension) 2D-patterned, chemically heterogeneous surfaces has been studied^22–24^.

For 1D (line/space) geometries, the wetting is inherently anisotropic, differing in the directions along (*θ*_∥_) and across (*θ*_⊥_) the pattern lines^16^. The liquid drop experiences a heightened energy barrier to spreading due to sharp-edge pinning in the direction perpendicular to the 1D lines while it is free to expand in the orthogonal direction as it would be on a uniform surface. Both the Cassie-Baxter and Wenzel Models were formulated for randomly textured 2D surfaces and are not immediately applicable to anisotropic 1D periodic patterns^20^. Most often these models have been applied to the contact angle perpendicular to the 1D lines (*θ*_⊥_). However, we are not aware of detailed discussion of the application of these models to predict drop characteristics in the 1D nanoscale regime important for evolving applications. Molecular dynamics simulations have focused on wetting on nanoscale groove-patterned surfaces. As a result of computational limitations, the simulations are limited to very small drops covering only a small number nm-scale line/space pairs^25–29^.

Many fabrication techniques have been used to prepare surfaces for wetting studies, including: interferometric lithography^14^. strained micro-wrinkling^16^, nano-imprint lithography^30,31^, and embossing^32^. Most investigations have been carried out for micrometer and larger scales, accessible by simple lithography approaches; only a few studies of nanoscale structures have been presented, and a systematic study with variation of pitch in the nanoscale regime has not been presented previously. Experimental studies of directional wetting on 1D patterned surface have been reported for large micro-scale features; to date nanoscale grooves, where microscopy has insufficient resolution to provide details of the drop/surface contact, have been relatively unexplored^15^.

The geometry of our sample is shown in Fig. 1b. The duty cycle (*DC*) is defined as the ratio of the wall thickness to the period (*x*/*p* = *x*/(*x* + *y*)); the spec cycle (*SC*) as the ratio between the width of the wall and the thickness of the photoresist/ARC stack (*SC* = *x*/*h*) The Cassie-Baxter model applied to the perpendicular contact angle is a function of the dimensions of the wall (*x*) and the cavity (*y*) of periodic structures (*p* = *x* + *y*) and is independent of *h*. The Wenzel model calculates the perpendicular contact angle as a function of the wall (*x*), cavity (*y*), and height (*h*) dimensions of the patterned structures^33^.

Both models start from Young’s equation for a smooth surface^34,35^,

1 $$\cos ({\theta }_{Y})=\frac{{\gamma }_{SV}-{\gamma }_{SL}}{{\gamma }_{VL}}$$

where γ_*SL*_, γ_*SV*_ and γ_*LV*_ are the free surface energies of the solid-liquid, solid-vapor and liquid-vapor interfaces, respectively, and the Young’s contact angle *θ*_*Y*_ is measured internal to the drop from the surface to the tangent to the liquid-vapor interface at the surface. For a blanket PR film under our experimental etching protocol, Young’s angle is 100°; for a blanket Silicon under our experiment protocol, Young’s angle is 95°^36^. In terms of the structure parameters, the key factor in the Wenzel model is the roughness factor *r* which is defined as the ratio of the contoured interfacial area to its planar projection, for our structure $r=\frac{x+y+2h}{x+y}=1+2\frac{h}{p}$ then

2 $$\cos \,{\theta }_{W}=r\,\cos \,{\theta }_{Y}=(1+2\frac{h}{p})\cos \,{\theta }_{Y}=(1+2\frac{h/x}{p/x})\cos \,{\theta }_{Y}=(1+2\frac{DC}{SC})\cos \,{\theta }_{Y}$$

For the Cassie-Baxter regime, where the liquid only contacts the top of the photoresist lines the contact angle is given by:

3 $$\cos \,{\theta }_{CB}=\frac{x}{p}[\cos ({\theta }_{Y})+1]-1=DC[\cos ({\theta }_{Y})+1]-1$$

Note that *θ*_W_ depends on both the *DC* and the *SC*. In contrast, *θ*_CB_ is independent of the PR height and the pattern period and depends only on the duty cycle (*DC*).

### Samples

A total of 32 samples equally divided in 2 sets as shown in Table 1 (fixed spec cycle) and Table 2 (fixed *h*) were investigated for each *DC* (only the 30% *DC* samples are shown; see Supplementary Material for the 40% samples). The varying parameter (*x* or *x*/*h*) is bolded in each set. Figure 1c shows side and top view (1000 nm period) of SEM images representing the profile of the samples.

**Table 1:** Structure Parameters 30% *DC*, *SC* fixed at 26%.

Period (*p*) µm	Wall (*x*) µm	Cavity (*y*) µm	Height (*h*) µm	Duty Cycle (*x*/*p*)	Duty Ratio (*x*:*y*)	Spec Cycle (*x*/*h*)	Spec Ratio (*x*:*h*)
**0**.**3**	**0**.**112**	0.188	**0**.**38**	37%	1:1.68	29%	1:3.4
**0**.**4**	**0**.**128**	0.272	**0**.**48**	32%	1:2.13	27%	1:3.8
**0**.**5**	**0**.**15**	0.35	**0**.**6**	30%	1:2.33	25%	1:4.0
**0**.**6**	**0**.**18**	0.42	**0**.**7**	30%	1:2.33	26%	1:3.9
**0**.**7**	**0**.**215**	0.485	**0**.**82**	31%	1:2.26	26%	1:3.8
**0**.**8**	**0**.**25**	0.55	**0**.**98**	31%	1:2.20	26%	1:3.9
**0**.**9**	**0**.**277**	0.623	**1**.**07**	31%	1:2.25	26%	1:3.9
**1**.**0**	**0**.**306**	0.694	**1**.**138**	31%	1:2.27	27%	1:3.7

**Table 2:** Structure Parameters 30% *DC*, h fixed at 0.73 µm.

Period (*p*) µm	Wall (*x*) µm	Cavity (*y*) µm	Height (*h*) µm	Duty Cycle (*x/p*)	Duty Ratio (*x:y*)	Spec Cycle (*x/h*)	Spec Ratio (*x:h*)
**0**.**3**	**0**.**11**	0.19	0.734	37%	1:1.73	**15%**	**1:6**.**7**
**0**.**4**	**0**.**131**	0.269	0.734	33%	1:2.05	**18%**	**1:5**.**6**
**0**.**5**	**0**.**162**	0.338	0.731	32%	1:2.09	**22%**	**1:4**.**5**
**0**.**6**	**0**.**188**	0.412	0.731	31%	1:2.19	**26%**	**1:3**.**9**
**0**.**7**	**0**.**203**	0.497	0.731	29%	1:2.45	**28%**	**1:3**.**6**
**0**.**8**	**0**.**231**	0.569	0.731	29%	1:2.46	**32%**	**1:3**.**2**
**0**.**9**	**0**.**263**	0.637	0.731	29%	1:2.42	**36%**	**1:2**.**8**
**1**.**0**	**0**.**294**	0.706	0.731	29%	1:2.40	**40%**	**1:2**.**5**

## Results and Discussion

**Figure 2:**
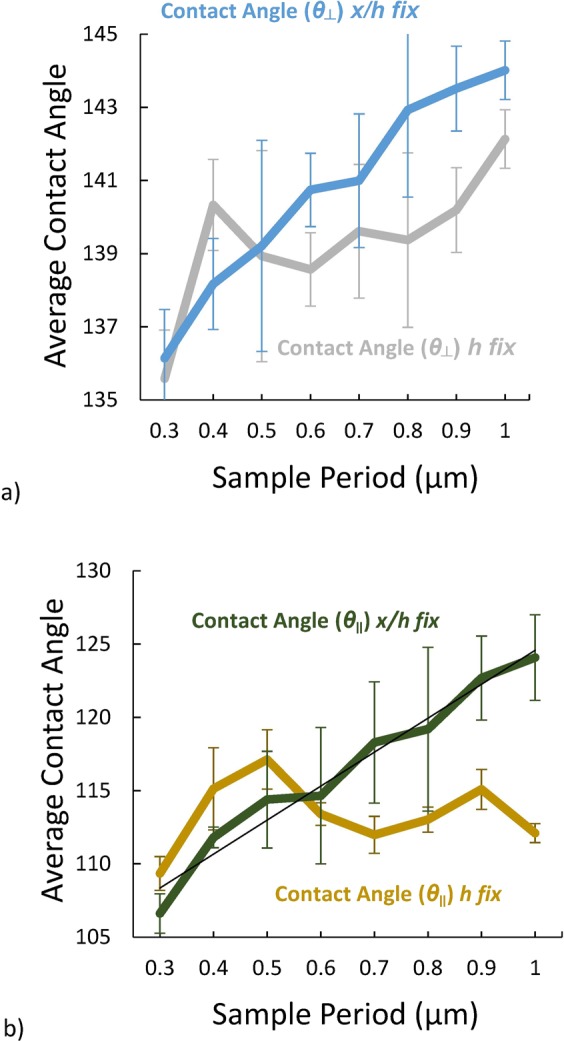
Variation of (**a**) *θ*_*⊥*_ and (**b**) *θ*_*||*_ for a 30% *DC* vs. period. Results are shown both for *x/h* fixed and *h* fixed.

Figure 1d shows the definitions of the experimentally measured drop parameters: *θ*_⊥_, *θ*_∥_, *L*, *W*. Figure 2 shows the measured *θ*_⊥_ and *θ*_∥_ contact angles as a function of period for both *x/h* fixed and *h* fixed. *θ*_||_ shows an increase with period when *x*/*h* is fixed and remains essentially independent of period for *h* fixed (Fig. 2a for *DC* = 30%). When the thickness of the photoresist is fixed, the contact angles (*θ*_||_ and *θ*_⊥_) remain constant independent of the period. On the other hand, the contact angles increase with period when the *SC* (*x*/*h*) is fixed at 26% (e.g. when the resist thickness increases along with the period). Moreover, the contact angles in the perpendicular direction are larger and show somewhat less variation from the lowest period (0.3 μm) to the highest period (1 μm). Note that as a result of experimental limitations (photoresist pattern collapse); the *DC* for the smallest 0.3 μm period with *h* and *x/h* fixed is somewhat larger than the desired 30% which probably accounts for the lower contact angles for *h* fixed at this period.

The *θ*_||_ and *θ*_⊥_ plots provide empirical dependencies for both perpendicular and parallel contact angles. For fixed 30% *DC* when *x*/*h* is fixed to 25% *SC* we can evaluate the contact angle as a function of period (experimental fit) by:

4 $$\begin{array}{cccc}\cos ({\theta }_{||}(p)) & \approx  & {m}_{1}\times p-0.705\, & {m}_{1}=-\,1.04\times {10}^{-4}\,{{\rm{n}}{\rm{m}}}^{-1}\\ \cos ({\theta }_{\perp }(p)) & \approx  & {m}_{2}\times p-0.266\, & {m}_{2}=-\,2.94\times {10}^{-4}\,{{\rm{n}}{\rm{m}}}^{-1}\end{array}$$

Similar results have been obtained for a 40% *DC* (data in Supplementary materials). For fixed 40% *DC* when *x/h* is fixed to 28% *SC*, we can empirically evaluate the contact angle as a function of period as:

5 $$\begin{array}{cccc}\cos ({\theta }_{||}(p)) & \approx  & {m}_{3}\times p-0.703\, & {m}_{3}=-\,9.2\times {10}^{-5}\,{{\rm{n}}{\rm{m}}}^{-1}\\ cos({\theta }_{\perp }(p)) & \approx  & {m}_{4}\times p-0.540\, & {m}_{4}=-\,1.16\times {10}^{-4}{{\rm{n}}{\rm{m}}}^{-1}\end{array}$$

Comparing with the model dependencies given in Eqs 2 and 3, it is clear, as expected, that neither model provides a satisfactory explanation of the data. Since the *DC* is fixed in both measurements, the Cassie Baxter model does not predict any variation in contact angles across period; however, there is a clear increase in contact angle for the *x/h* fixed case. For the Wenzel model, the contact angles should be fixed for a fixed *SC* (*x/h*) along with a fixed *DC* (*x*/*p*) and vary for a fixed *h*, the opposite of the observation.

In order to show the pinning behavior on the perpendicular direction (*θ*_⊥_), advancing and receding contact angles were measured on two samples (300 nm and 900 nm pitch). For the 300 nm pitch sample, the advancing angle was measured to be 145.7° and the receding angle 122.0° For the 900 nm pitch sample, the advancing angle was measured to be 158.1°, and the receding angles was measured to be 137.1°. Table 3 and 4 shows all measured values. Clearly, the perpendicular direction has a much larger variation due to the pinning of the droplet on the pattern walls. Also, it is noticeable that for the parallel direction the variation is low due to the free position of the droplet in the orthogonal direction of the walls.

**Table 3:** Pinning Experimental Results for 300 nm and 900 nm.

	*θ*_⊥_ Advancing	*θ*_⊥_ Receding	*θ*_||_ Advancing	*θ*_||_ Receding
300 nm	145.7°	121.95°	122.6°	120.8°
900 nm	158.1°	137.1°	145.5°	144.6°

**Table 4:** IL Exposure/develop parameters.

Period (μm)	Total Exposure Dose (mJ/cm^2^)	Develop time (s)
0.3	96.9	20
0.4	104.0	25
0.5	111.2	30
0.6	114.8	30
0.7	118.4	30
0.8	121.9	30
0.9	125.6	30
1.0	129.1	30

Developer type: MF-26, hard bake temperature was 110 °C for 60 s for all samples. Total exposure dose was the same for both fixed *h* and fixed *x/h* samples.

**Figure 3:**
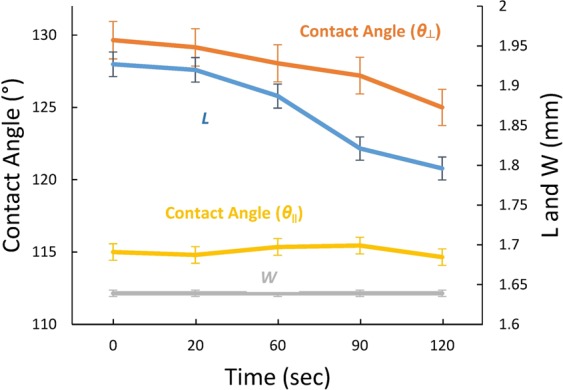
Width (*W*), Length (*L*), *θ*_⊥_ and *θ*_||_ as a function of time for 0.3 µm pitch for a 30% *DC* sample showing the variation as the drop dries.

To further characterize the pinning effect, a 4 μL DI water droplet was placed on the surface of the 300 nm sample at 38% humidity for 2 minutes. Five different measurements were obtained on one sample in order to assess the standard deviation. As the drop evaporated over time, we measured both contact angles (*θ*_⊥_ and *θ*_||_), *L*, and *W*. The *θ*_⊥_ slowly reduces over time due to pinning while *θ*_||_ remains constant. Similarly, the width of the drop stays constant while the length of the drop decreases over time; clearly exhibiting pinning on the walls in the transverse direction and the free motion of the interface in the parallel direction as shown in Fig. 3.

**Figure 4:**
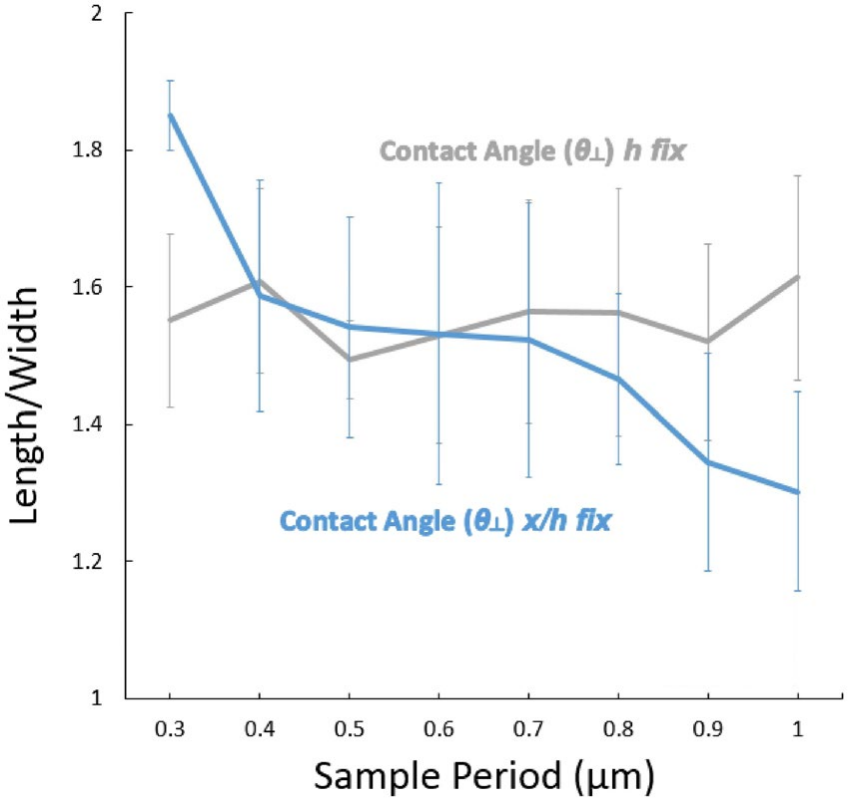
*L*/*W* as a function of period (0.3–1 μm) for 30% *DC* samples. Drop Volume nominally = 4 μL.

Figure 4 shows the measured *L*/*W* ratio as the period is varied (fixed *DC*, both *h*-fixed and *x*/*h*-fixed results). At smaller periods, the *L*/*W* ratio is larger at higher periods for *x*/*h* fixed and is independent of period for *h*-fixed; the *h*-fixed results are consistent with a Cassie-Baxter model where the liquid does not penetrate the gaps between PR lines and so is independent of *h* for a fixed *DC*; however, the *x*/*h* fixed results show the drop tending toward equal length and width (e.g. becoming less elliptical and more circular) as the period increases.

**Figure 5:**
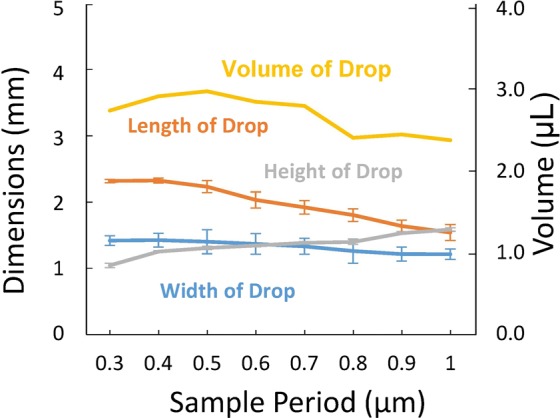
Width (*W*), Height (*h*), and Length (*L*) as a function of period (0.3–1 µm) for 30% *DC* samples. The calculated volume is also shown.

The ratio of drop length and drop width (*L/W*) determines the shape of the droplet. Since higher contact angles (only when *x/h* is fixed) are obtained for higher periods the drop sits differently; the larger the period, the more circular the shape of the drop. The insets show top-down micrographs of the drops for periods of 0.5 and 1.0 µm. Figure 5 shows the width, height, and length of the drop as a function of period. Drop volume was calculated with Eq. 6 which was developed for elongated droplets as observed from the top^37^.

6 $$V(h,W,L)=[(h\ast W)-(4-\pi ){(\frac{2}{h}+\frac{2}{W})}^{-2}]\times (L-\frac{W}{3})$$

Figure 5 shows the width, height, and length of the drop as a function of period. Drop volume was calculated with Eq. 6 which was developed for elongated droplets observed from a top view image. The calculated volume as a function of length, height, and width of the drop is shown in Fig. 5; some of the drop liquid was left in the syringe reducing the nominal drop volume of 4 µL. As required by the experimental protocol, the volume of the drop was fixed over the variation of period.

## Conclusions

**Figure 6:**
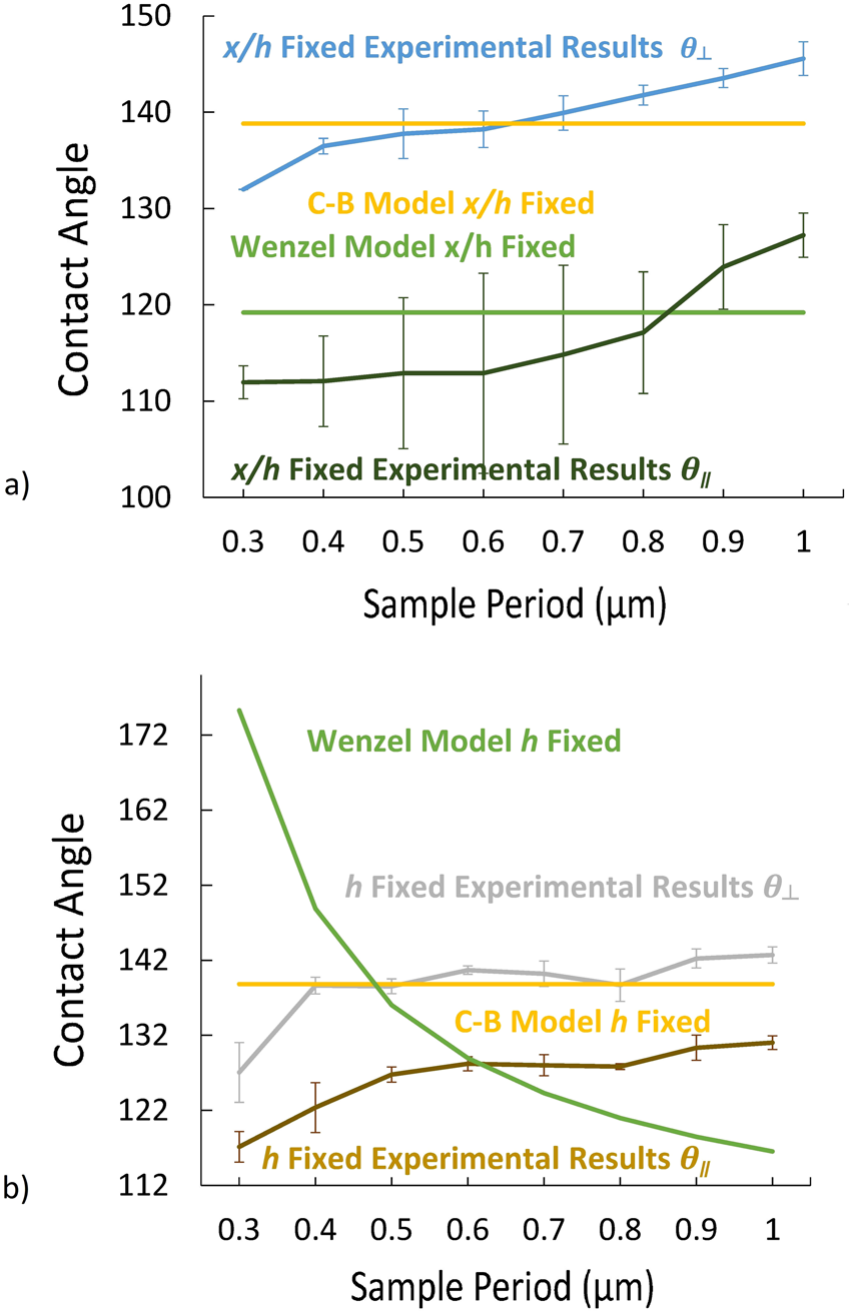
Contact angle comparison (*θ*_*⊥*_, *θ*_*||*_). Experiment vs. W and C-B models: (**a**) *x*/*h* fixed; and (**b**) *h* fixed.

The drop parameters (contact angles and dimensions) have been investigated for DI water drops atop nanoscale 1D photoresist lines on Si substrates as a function of period across the range 0.3- to 1.0-µm with a common plasma etch treatment to assure similar interaction parameters. The drops are elongated, reflecting the 1D patterned substrate and neither Cassie-Baxter nor Wenzel models can explain the dependence of the contact angles and length/width ratio as the period is changed with either a constant photoresist height or a constant photoresist width/height ratio. Figure 6 shows comparison of experimental data vs. Cassie-Baxter and Wenzel’s models. For *x*/*h* fixed, both models predict constant contact angles; experimentally, both parallel and perpendicular angles increase as the period is increased. For *h* fixed, the Wenzel model predicts decreasing contact angles while the Cassie-Baxter model predicts a constant contact angle; experimentally, the perpendicular contact angle is relatively constant while the parallel contact angle increases as the period is increased. More detailed modeling is necessary to provide a full understanding of the static and dynamic behavior of liquid drops on 1D patterned surface.

More specifically, the experiments show significant standard deviation in the measurements in some regimes. Clearly, such a distribution suggests a significant dependence on the dynamics of the drop deposition on the sample. Interestingly, due to the pinning effect one would expect that the uncertainty in drop length in the perpendicular direction, and the drop contact angle for that matter should be larger than in the parallel direction. The data does not show this, however, because the volume of the drop remains constant, and so any measured uncertainty in the one direct will manifest similarly in the other. To quantify the extent of the pinning and how it affects measurement uncertainty, capillary hydrostatic simulations could be pursued with the Surface Evolver^38^. Pinning on a “sharp” corner cannot be observed in the experiment and leads to a measured “apparent” angle macroscopically. Simulations could quantify this effect. In 3D, or even in a simpler axi-symmetric geometry, capillary hydrodynamics models of the sort in Baer *et al*.^39^ could be pursued to understand better the way in which the drop comes to equilibrium and to quantify the sensitivity to drop detachment and settling. The Bond number (which measures the importance of gravitational forces to surface tension forces in all cases is less than one but not by much, (of order 0.3), and so measurement aberrations due to surface distortion could impact the results. Capillary waves as a result of inertia/ringing when the drop detaches from the syringe would also effect the final resting hydrostatic shape. All these phenomena would require a full capillary hydrodynamic model to better understand their effects.

These results show that the drop shape provides a sensitive macroscopic metrology approach that is sensitive to the nanoscopic features of the surface. Particularly for a process monitoring application where the drop dimensions are measured on multiple samples moving through a manufacturing process, the drop measurements provide a simple early warning for process control issues without requiring nanoscopic measurements.

## Methods

### Sample Fabrication

We used interferometric lithography to create periodic patterns due to its advantages: low cost, large area capability, short exposure time, simplicity, and flexibility^14^. Samples were cleaned with a Piranha treatment with a ratio of 1:3 of 30% Hydrogen Peroxide (H_2_O_2_) and 98% Sulfuric Acid (H_2_SO_4_) followed by an HF dip in a solution with a ratio of 1:3 of 49% Hydrofluoric Acid and deionized Water (DI H_2_O) to remove contamination and native oxide layers from the silicon surface. After cleaning, a layer of bottom anti-reflective coating (BARC, iCON7), is spun-on and hot-plate baked (205 °C, 60 sec) to minimize reflections from the silicon surface during the lithography step. Following the bake, a layer of positive photoresist (SPR505) is spin-coated atop the BARC and hot-plate prebaked (90 °C, 90 sec). Interferometric lithography (IL) with a 355-nm frequency tripled YAG laser (Coherent Model Infinity 40–100) was used to fabricate all 1D nano-periodic samples. The pattern period variation from 0.3- to 1-μm was achieved by changing the IL angle of incidence; samples with a 30% duty cycle were obtained by controlling the total dose and develop conditions, as shown in Table 4. The thickness of the photoresist was controlled by varying spin speed during deposition and was calculated based on achieving either an *SC* of 26% and a *DC* of 30% or a fixed *h* of 0.73 µm with a fixed 30% *DC* for different periods. All samples were exposed to a reactive ion etch (RIE) with CHF_3_ with a flow rate of 8mTorr of rough pressure, for 20 seconds with an RF power of 45 W. This procedure removed the BARC layer between the photoresist lines while minimally affecting the widths of the photoresist lines and also provided a consistent chemical surface potential for the photoresist surface^34^. A Plasma-Lab RIE Etcher Model ACG-3. Was used All of the structures were characterized by SEM at 20 kV as shown in Fig. 1c.

### Contact Angle Measurement

All contact angles, width, length, and ratios were measured with an AST Products, Inc VCA Optima Contact Angle Instrument tool with a 4 µL drop volume. For each parameter variation, we fabricated two samples and averaged five measurements on each to find both the average value and the statistics of the measurement. SEM was used to characterize (cross section and top-down views) the samples

## Supplementary information

Dataset 1 (40% DC)

## References

[1] Kitsomboonloha R, Morris SJS, Rong X, Subramanian V (2012). Femtoliter-scale patterning by high-speed, highly scaled inverse gravure printing. Langmuir.

[2] Schmid, G. M. *et al*. Jet and ash imprint lithography for the fabrication of patterned media drives. *Proc*. *SPIE*. Photomask Technology **7488**, 748820 (2009).

[3] Dussan EB (1979). On the spreading of liquids on solid surfaces: static and dynamic contact lines. Ann. Rev. Fluid Mech..

[4] Woodward JT, Gwin H, Schwartz DK (2010). Contact angles on surfaces with mesoscopic chemical heterogeneity. Langmuir.

[5] Marmur A (1996). Equilibrium contact angles: theory and measurement. Colloids & Surfaces A.

[6] Palasantzas G, De Hosson J, Th M (2001). Wetting on rough surfaces. Acta Materialia.

[7] Patankar NA (2003). On the modeling of hydrophobic contact angles on rough surfaces. Langmuir.

[8] Bormashenko E, Musin A, Whyman G, Zinigrad M (2012). Wetting transitions and depinning of the triple line. Langmuir.

[9] Feng L (2002). Super-hydrophobic surfaces from natural to artificial. Adv. Mater..

[10] Nosonovsky M (2007). On the range of applicability of the Wenzel and Cassie equations. Langmuir.

[11] Cassie ABD, Baxter S (1944). Wettability of porous surfaces. Trans. Faraday Soc..

[12] Wenzel RN (1936). Resistance of solid surfaces to wetting by water. Ind. Eng. Chem..

[13] Park SG, Moon JH, Jeon HC, Yang SM (2012). Anisotropic wetting and superhydrophobicity on holographically featured 3D nanostructured surfaces. Soft Matter.

[14] Xia D, Ku Z, Lee SC, Brueck SRJ (2011). Nanostructures and functional materials fabricated by interferometric lithography. Adv Mater..

[15] Xia D, Brueck SRJ (2011). Strongly anisotropic wetting on one-dimensional nanopatterned surfaces. Nano Lett..

[16] Xia D, Johnson LM, Lopez GP (2012). Anisotropic wetting surfaces with one-dimensional and directional structures: Fabrication approaches, wetting properties and potential applications. Adv. Matls..

[17] Wang Z, Zhao Y-P (2017). Wetting and electrowetting on corrugated substrates. Phys. Fluids.

[18] Kim (2016). Controlled Anisotropic Wetting of scalloped Silicon Nanogroove. RSC.

[19] Chung JY, Youngblood JP, Stafford CM (2007). Anisotropic Wetting on Tunable Micro-wrinkled Surfaces. Soft Matter.

[20] Neuhaus S, Spencer ND, Padeste C (2012). Anisotropic wetting of microstructured surfaces as a function of surface chemistry. ACS Appl. Mater. Interfaces.

[21] Weng Y-H, Hsieh F, Tsao H-K, Sheng Y-J (2017). Water-repellent Hydrophilic Nanogrooves. RSC.

[22] Larsen ST, Taboryski R (2008). A Cassie-like law using triple phase boundary line fractions for faceted droplets on chemically heterogeneous surfaces. Langmuir.

[23] Cubaud T, Fermigier M (2001). Faceted drops on heterogeneous surfaces. Europhys. Lett..

[24] Courbin L (2007). Imbibition by polygonal spreading on microdecorated surfaces. Nature Materials.

[25] Yong X, Zhang LT (2009). Nanoscale Wetting on groove-patterned surfaces. Langmuir.

[26] David R, Neumann AW (2012). Shapes of drops in the Cassie state on grooved surfaces. Colloids and Surfaces..

[27] Shahraz A, Borhan A, Fichthorn KA (2013). Wetting on physically patterned solid surfaces: the relevance of molecular dynamics simulations to macroscopic systems. Langmuir.

[28] Khan S, Singh JK (2013). Wetting Transition on nanodroplets of water on textured surfaces: a molecular dynamics study. Mol. Simul..

[29] Chen S, Wang J, Chen D (2014). States of a water droplet on nanostructured surfaces. J. Phys. Chem..

[30] Guder F, Yang Y, Kruger M, Stevens GB, Zacharias M (2010). Atomic layer deposition on phase-shift lithography generated photoresist patterns for 1D nanochannel fabrication. ACS Appl. Mater. Interfaces.

[31] Yu Z, Gao H, Wu W, Ge H, Chou S (2003). Fabrication of large area subwavelength antireflection structures on Si using trilayer resist nanoimprint lithography and liftoff. J. Vac. Sci. Technol..

[32] Kusumaatmaja H, Vrancken RJ, Bastiaansen CWM, Yeomans JM (2008). Anisotropic drop morphologies on corrugated surfaces. Langmuir.

[33] Duta, L., Popescu, A. C., Zgura, I., Preda, N. & Mihailescu, I. N. Wettability of nanostructured surfaces. *INTECH*, 207–251 (2015).

[34] Nosonovsky M (2007). On the range of applicability of Wenzel and Cassie equations. Langmuir.

[35] Gao L, McCarthy TJ (2009). Wetting 101. Langmuir.

[36] Xia D, He X, Ziang Y-B, Lopez GP, Brueck SRJ (2010). Tailoring anisotropic wetting properties on submicrometer-scale periodic grooved surfaces. Langmuir.

[37] Musterd M, Volkert VS, Kleijn CR, Kreutzer MT (2015). Calculating the volume of elongated bubbles and droplets in microchannels from a top view image. Royal Society of Chem..

[38] Brakke KA (1992). The Surface Evolver. Experimental Mathematics.

[39] Baer TA, Schunk PR, Cairncross RA, Rao RR, Sackinger PA (2000). A finite element method for free surface flows of incompressible fluids in three dimensions. Int. J. Numer. Meth. Fluids..

