# Supplementary material for: Measuring Liquid Drop Properties on Nanoscale 1D Patterned Photoresist Structures
Source: Sci Rep. 2019 Apr 5;9:5723. doi: 10.1038/s41598-019-42106-z (PMC6450940; doi:10.1038/s41598-019-42106-z)
Supplement: Supplementary file 1 — Dataset 1 (40% DC) [file 41598_2019_42106_MOESM1_ESM.docx]

**Measuring Liquid Drop Properties on Nanoscale
1D Patterned Photoresist Structures**

**Juan J. Faria-Briceno^1^, Alexander Neumann^1^, P. Randall Schunk^2^ and S. R. J. Brueck^1^**

**Supplementary Results for 40% DC for both *h-*fixed and *x/h-* fixed**

Figure S1 presents the experimentally measured contact angles: *θ*_⊥_*,* and *θ*_||_ as a function of period variation from 300 nm to 1000 nm for both *x/h* fixed and *h* fixed for a 40% duty cycle. Similarly to 30% DC results, *θ*_||_ shows an increase with period when *x*/*h* is fixed and remains essentially independent of period for *h* fixed. As shown in Table SI, at 300 nm, the fabrication limitations (pattern collapse) resulted in a larger DC (45%) which accounts for the deviation from a nominally constant contact angle.

| **Table SI: Structure Parameters 40% DC, SC fixed** | | | | | | | |
| --- | --- | --- | --- | --- | --- | --- | --- |
| Period (*p*) um | Wall (*x*) um | Cavety (*y*) um | Height (*h*) um | Duty Cycle (*x/p*) | Duty Ratio (*x:y*) | Spec Cycle (*x/h*) | Spec Ratio (*x:h*) |
| **0.3** | **0.135** | 0.165 | **0.38** | 45% | 1:1.22 | 36% | 1:2.8 |
| **0.4** | **0.149** | 0.251 | **0.48** | 37% | 1:1.68 | 31% | 1:3.2 |
| **0.5** | **0.19** | 0.31 | **0.6** | 38% | 1:1.63 | 32% | 1:3.2 |
| **0.6** | **0.23** | 0.37 | **0.7** | 38% | 1:1.61 | 33% | 1:3.0 |
| **0.7** | **0.286** | 0.414 | **0.82** | 41% | 1:1.45 | 35% | 1:2.9 |
| **0.8** | **0.3** | 0.5 | **0.98** | 38% | 1:1.67 | 31% | 1:3.3 |
| **0.9** | **0.354** | 0.546 | **1.07** | 39% | 1:1.54 | 33% | 1:3.0 |
| **1** | **0.394** | 0.606 | **1.138** | 39% | 1:1.54 | 35% | 1:2.9 |

| **Table SII: Fabrication Parameters 40% DC *h* fixed at 0.79 µm** | | | | | | | |
| --- | --- | --- | --- | --- | --- | --- | --- |
| Period (*p*) um | Wall (*x*) um | Cavety (*y)* um | Height (*h)* um | Duty Cycle (*x/p*) | Duty Ratio (*x:y*) | Spec Cycle (*x/h*) | Spec Ratio (*x:h*) |
| **0.3** | **0.138** | 0.162 | 0.734 | 46% | 1:1.17 | **19%** | **1:5.3** |
| **0.4** | **0.155** | 0.245 | 0.734 | 39% | 1:1.58 | **21%** | **1:4.7** |
| **0.5** | **0.206** | 0.294 | 0.79 | 41% | 1:1.43 | **26%** | **1:3.8** |
| **0.6** | **0.225** | 0.375 | 0.79 | 38% | 1:1.67 | **28%** | **1:3.5** |
| **0.7** | **0.281** | 0.419 | 0.79 | 40% | 1:1.49 | **36%** | **1:2.8** |
| **0.8** | **0.3** | 0.5 | 0.79 | 38% | 1:1.67 | **38%** | **1:2.6** |
| **0.9** | **0.363** | 0.537 | 0.79 | 40% | 1:1.48 | **46%** | **1:2.2** |
| **1** | **0.388** | 0.612 | 0.79 | 39% | 1:1.58 | **49%** | **1:2.0** |

Fig. S1: Variation of a) *θ_⊥_* and b) *θ_||_* for a 40% *DC* vs. period. Results are shown both for *x/h* fixed and *h* fixed.

a)

b)

Figure S2 shows the measured length/width (*L*/*W*) ratio as the period is varied (fixed 40% DC, both *h*-fixed and *x*/*h*-fixed results). At smaller periods, the *L*/*W* ratio is larger than at higher periods for *x*/*h* fixed and is independent of period for *h*-fixed; the *h*-fixed results are consistent with a Cassie-Baxter model where the liquid does not penetrate the gaps between PR lines and so is independent of *h* for a fixed DC; however, the *x*/*h* fixed results show the drop tending toward equal length and width (e.g. becoming less elliptical and more circular) as the period increases while it would be independent of *x*/*h* for a Cassie-Baxter model.

Fig. S2: *L*/*W* as a function of period (0.3-1 μm) for 40% *DC* samples.
